# Timing and motivation behind the decision to become a surgeon: Gender and optimal recruitment insights

**DOI:** 10.1007/s00595-026-03361-6

**Published:** 2026-06-15

**Authors:** Junko Mukohyama, Genki Watanabe, Yoshiyuki Kiyasu, Daisuke Koike, Jun Watanabe, Saseem Poudel, Atsushi Tanikawa, Takashi Kohmura, Yoshiko Yamaoka-Fujikawa, Keiko Hosoya, Hiroko Watayo, Mitsue Saito, Norihiko Ikeda, Akinobu Taketomi

**Affiliations:** 1 Japan Surgical Society Education Committee, Japan Surgical Society, Tokyo, Japan; 2 Division of Frontier Surgery, The Institute of Medical Science, The University of Tokyo, Tokyo, Japan; 3 Department of Surgery, The Institute of Medical Science, University of Tokyo, 4-6-1 Shirokanedai, Minato-ku, Tokyo, 108-8639 Japan; 4 Department of Hepatobiliary-Pancreatic Surgery, Graduate School of Medicine, Osaka Metropolitan University, 1-4-3 Asahimachi, Abeno-ku, Osaka, Japan; 5 Department of Surgery, Kyoto University Graduate School of Medicine, Kyoto, Japan; 6 Department of Gastroenterological Surgery, Fujita Health University School of Medicine Bantane Hospital, Aichi, Japan; 7 Department of Patient Safety, Nagoya University Hospital, Nagoya, Japan; 8 Department of Surgery, Division of Gastroenterological, General and Transplant Surgery, Jichi Medical University, Tochigi, Japan; 9 Department of Gastroenterological Surgery II, Hokkaido University Graduate School of Medicine, Sapporo, Japan; 10 Department of Emergency and Critical Care Medicine, Tohoku University Hospital, Sendai, Japan; 11 Department of Surgery, International University of Health and Welfare Atami Hospital, Shizuoka, Japan; 12 Department of Upper Gastrointestinal Surgery, Kitasato University School of Medicine, Kanagawa, Japan; 13 Department of Breast and Endocrine Surgery, Tottori University Hospital, Tottori, Japan; 14 Department of Pediatric General and Urogenital Surgery, Juntendo University School of Medicine, Tokyo, Japan; 15 Department of Breast Oncology, Juntendo University Graduate School of Medicine, Tokyo, Japan; 16 Japan Surgical Society, Tokyo, Japan; 17 Department of Surgery, Tokyo Medical University, Tokyo, Japan; 18 Department of Gastroenterological Surgery I, Hokkaido University Graduate School of Medicine, Hokkaido, Japan

**Keywords:** Timing and motivation, Career decision, Surgical career, Surgical residency training

## Abstract

**Purpose:**

This study aimed to clarify the timing and motivations behind decisions to pursue a surgical career and identify optimal recruitment strategies.

**Methods:**

A nationwide questionnaire was administered to newly certified surgical trainees. Respondents were classified into two groups based on when they decided to pursue surgery: before junior residency (BJR) or during/after junior residency (DJR). The backgrounds and motivations were compared.

**Results:**

Among the 758 respondents (53.8%), 25.6% were female. Male trainees were more likely to decide to pursue surgery before medical school (18.7% vs. 9.4%, *p* = 0.003), whereas female trainees more often decided during their PGY-2 (35.4% vs. 24.1%, *p* = 0.002). The DJR group included more women (30.3% vs. 21.7%, *p* = 0.014) and residents from urban areas (46.6% vs. 36.7%, *p* = 0.008). The BJR group was more influenced by the images of surgeons and family. Among female residents, those in the DJR group were more influenced by recruitment from the surgeons.

**Conclusions:**

This study demonstrated sex differences in the timing and motivation for pursuing a surgical career. Understanding the optimal timing and appealing factors for recruiting surgeons, particularly women, could inform more effective recruitment strategies.

**Supplementary Information:**

The online version contains supplementary material available at https://doi.org/10.1007/s00595-026-03361-6.

## Introduction

Japanese society is currently facing a serious shortage of surgeons [1]. According to the statistics of physicians, dentists, and pharmacists reported by the Ministry of Health, Labor and Welfare, the number of surgeons who identified their primary specialty as general surgery, gastrointestinal surgery, cardiovascular surgery, thoracic surgery, breast surgery, or pediatric surgery decreased from 28,361 in 2000 to 23,073 in 2022 [2, 3]. The decline in the number of surgeons is attributed to multiple interrelated factors, including excessive working hours, heavy workloads, risk of medical litigation, imbalance between compensation and workload, and an unsupportive working environment for female physicians [4–6].

The decision to pursue a surgical career is shaped by a complex interplay of personal aspirations, professional ideals, and broader social influences. Despite ongoing advancements in surgical training and increasing attention to work–life balance, attracting and retaining young physicians in the surgical field remains a global challenge. A study conducted in the United States in 2016 reported that 62% of surgeons were fairly certain about pursuing surgery before entering medical school, 13% decided during their preclinical years, and 25% during their clerkship years, whereas the corresponding rates among non-surgeons were 40%, 7%, and 54%, respectively, indicating that surgeons tend to decide on their career path earlier [7]. In Japan, a survey conducted by the Japanese Medical Society Board found that 35.2% of respondents chose to become surgeons before graduating from medical school [8], indicating a slightly later decision than that in the U.S. study.

Factors such as perceptions of workload, career flexibility, and access to mentorship can influence the timing and motivation for choosing surgery. Individual experiences and expectations may vary according to demographic and personal backgrounds, including gender differences. Exploring these variations can help clarify potential barriers and inform supportive measures for aspiring surgeons. In Japan, after completing six years of undergraduate medical education, surgical training programs require medical graduates to complete a two-year structured postgraduate clinical training program. Subsequently, aspiring surgeons undergo at least a three-year surgical residency, after which they are eligible to take the Board of Surgery certification examination. In the present study, we performed a secondary analysis of a previous questionnaire survey conducted among recently board-certified surgeons [9] to examine sex differences in the timing and motivation for choosing a surgical career. These findings have direct implications for developing more effective surgeon recruitment strategies and sustaining a diverse future surgical workforce.

## Methods

### Subjects of this study

This study reports findings from an additional analysis of a previously reported nationwide online questionnaire survey targeting newly certified surgical trainees in 2023 [9–11]. An online survey containing 43 questions about the educational and work environments during surgical residency training was sent to the residents after their board-certification examination. All residents provided informed consent to participate in this survey and were informed of their right to refuse to respond in advance. The details of the questions and a summary of the results have been published previously [9]. This study was approved by the Institutional Review Board of the Japanese Surgical Society (JSS) (approval no. JSS2023-1).

### Overview of medical education and decision to pursue a surgical career in Japan

**Fig. 1:**
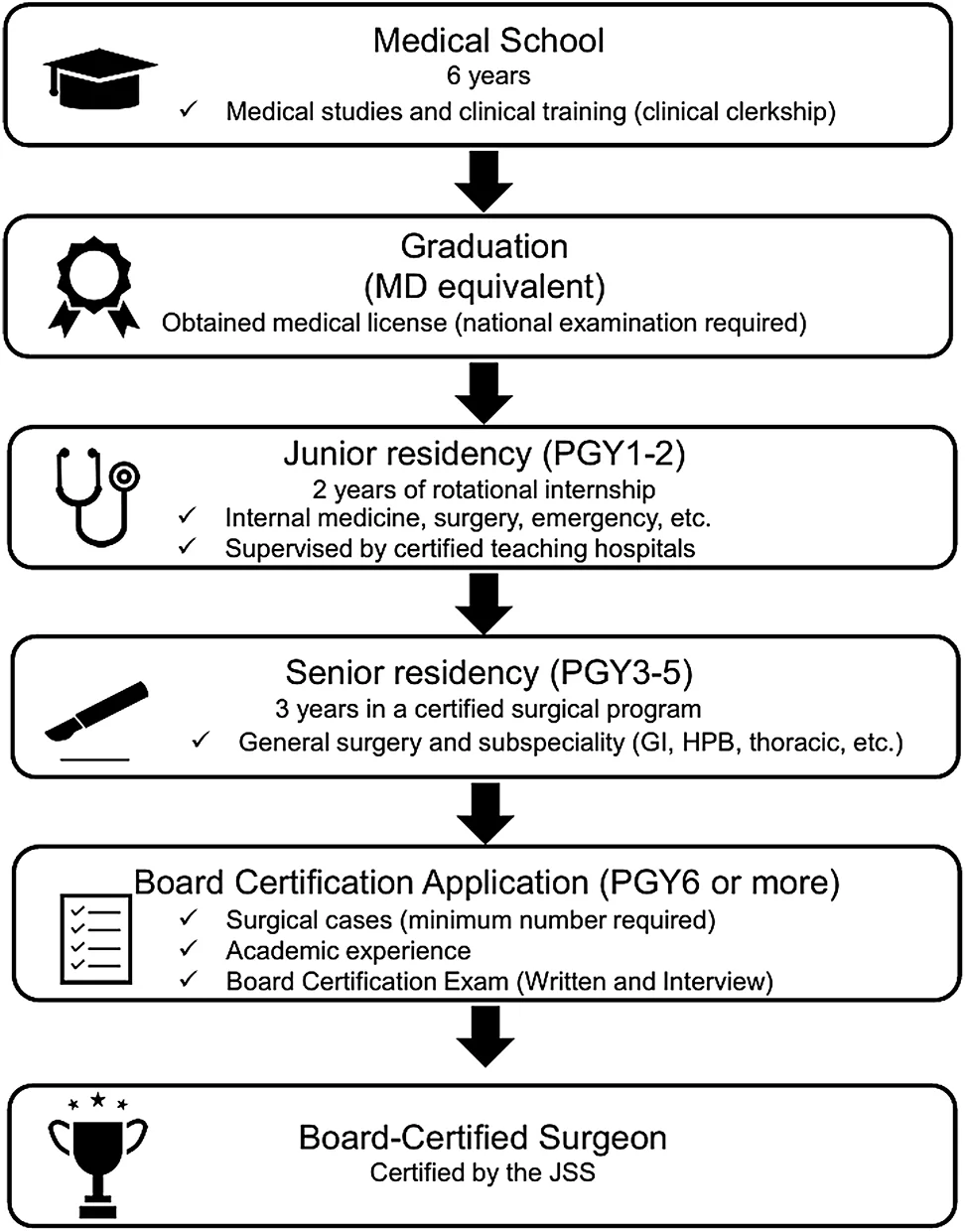
Career path of a board-certified surgeon in Japan. A schematic representation of the typical stages in the path to becoming a board-certified surgeon in Japan. After completing six years of medical school and passing the national licensing exam, physicians undergo two years of junior residency, followed by three years of senior residency in a certified program. Board certification requires surgical experience, academic activities, and passing both written and interview examinations. MD, Doctor of Medicine; PGY, postgraduate year; GI, gastrointestinal; HPB, hepato-pancreatic-biliary; JSS, Japan Surgical Society

A typical career path for surgeons in Japan is shown in Fig. 1. The path to becoming a doctor typically involves six years of medical school followed by two years of postgraduate clinical training after obtaining a medical license [12]. During the fourth to sixth years of medical school, medical students receive clinical training in university hospitals, known as clinical clerkship. After completing six years of medical education and passing a written examination, they are granted a medical license by the national authority. They then undergo a two-year clinical internship as junior residents. Choosing a specialty can be done anywhere from before medical school until the end of the internship period. Residents who decide to pursue a surgical career enter senior residency training, which is regulated by the JSS and requires a minimum of three years of surgical training, followed by passing the board certification examination in surgery. The present cohort consisted of newly board-certified surgical trainees in 2023, and Fig. 1 illustrates the typical training pathway relevant to this cohort within the current Japanese postgraduate training framework.

### Group definition to identify timing and motivation behind the decision to become a surgeon

The online survey asked residents about the timing of their surgical career decisions: before medical school, during medical school (before, during, or after clinical clerkship), during junior residency in post-graduate year (PGY)-1 or PGY-2, or during senior residency. Additionally, residents were asked to indicate whether they were positively, neutrally, or negatively influenced by eight major factors in deciding on their surgical career. These factors included surgery and procedures, the image of surgeons, recruitment from surgeons, educational system and process of becoming an independent surgeon, family or relatives, career path, salary, quality of life (QOL), and work-life balance (WLB). Respondents’ hometowns were categorized according to city size as follows: urban area (> 1 million population), regional city (50,000–1 million), and rural area (< 50,000).

To determine the optimal timing for recruiting surgeons during medical school or residency and to explore gender differences in pursuing a surgical career, residents were divided into two groups based on when they chose to pursue surgery: those who decided before junior residency (BJR) and those who decided during or after junior residency (DJR). The personal backgrounds and motivations for pursuing a surgical career were compared between the two groups and stratified by gender.

### Statistical analysis

While categorical variables were compared using the χ^2^ test or Fisher’s exact test, continuous variables were shown as median (range) and were compared using the Mann–Whitney *U* test. Factors that had a positive, neutral, or negative impact on deciding to pursue a surgical career were quantified in this order and compared between BJR and DJR using the Mann–Whitney *U* test. Although questions regarding the satisfaction level were evaluated using a 4-level Likert scale (extremely satisfied, moderately satisfied, moderately dissatisfied, and extremely dissatisfied), the *p-*value was calculated on two levels: satisfied and dissatisfied. In the tables, when the sum of the numbers for each variable does not match the BJR group (*n* = 406) or the DJR group (*n* = 343), this indicates that there are non-respondents. All tests were two-tailed, and *p* < 0.05 was considered statistically significant. Statistical analyses were conducted using IBM SPSS Statistics software program Version 28 (IBM Corporation, Armonk, NY, USA).

## Results

### Demographics of responding surgical residents

The response rate was 53.8% (758 out of 1410), of whom 194 (25.6%) were women surgeons. Of the 758 respondents, 406 (53.6%) were classified into the BJR group and 343 (45.3%) into the DJR group; the remaining respondents had missing data for the timing-of-decision item and therefore could not be classified into either group. The DJR group had a higher percentage of women (*n* = 104, 30.3%, *p* = 0.014) and surgeons from urban areas (*n* = 160, 46.6%, *p* = 0.008) than the BJR group (Table 1). There were no significant differences between the two groups in terms of post-graduate year, age, or subspecialty of interest. Satisfaction with the instructors’ clinical skills, educational skills, and the surgical residency training program was comparable between the groups.

**Table 1:** Demographics and evaluation of surgical residency training among responding surgical residents

Variables	BJR group (*n* = 406)	DJR group (*n* = 343)	*p*
Post-graduation, years			0.137
6	174 (42.9)	140 (40.8)	
7	184 (45.3)	145 (42.3)	
Other	48 (11.8)	58 (16.9)	
Age, years			0.382
≤30	70 (17.2)	47 (13.7)	
31–35	301 (74.1)	263 (76.7)	
36–40	31 (7.6)	26 (7.6)	
>40	4 (1.0)	7 (2.0)	
Sex, female	88 (21.7)	104 (30.3)	0.014
Hometown			0.008
Urban city	149 (36.7)	160 (46.6)	
Regional city	204 (50.2)	155 (45.2)	
Rural area	53 (13.1)	28 (8.2)	
Subspecialty of interest			0.174^a^
Gastroenterological surgery	203 (50.0)	189 (55.1)	
Cardiovascular surgery	64 (15.8)	22 (6.4)	
Thoracic surgery	49 (12.1)	36 (10.5)	
Breast surgery	35 (6.7)	50 (14.6)	
Other	55 (13.5)	46 (13.4)	
Considering drop out during surgical residency training, yes	252 (65.1)	222 (68.1)	0.401
Satisfaction with instructors’ clinical skills, yes	382 (96.0)	328 (97.6)	0.213
Satisfaction with instructors’ educational skills, yes	325 (81.7)	292 (86.9)	0.053
Satisfaction with surgical residency training program, yes	337 (84.7)	283 (84.5)	0.942

Data are shown as n (%). ^a^*P*-values were calculated for gastroenterological surgery or others

BJR: residents who chose a surgery career before junior residency. DJR: residents who chose a surgery career during or after junior residency

### Timing of decision to pursue a surgical career

**Fig. 2:**
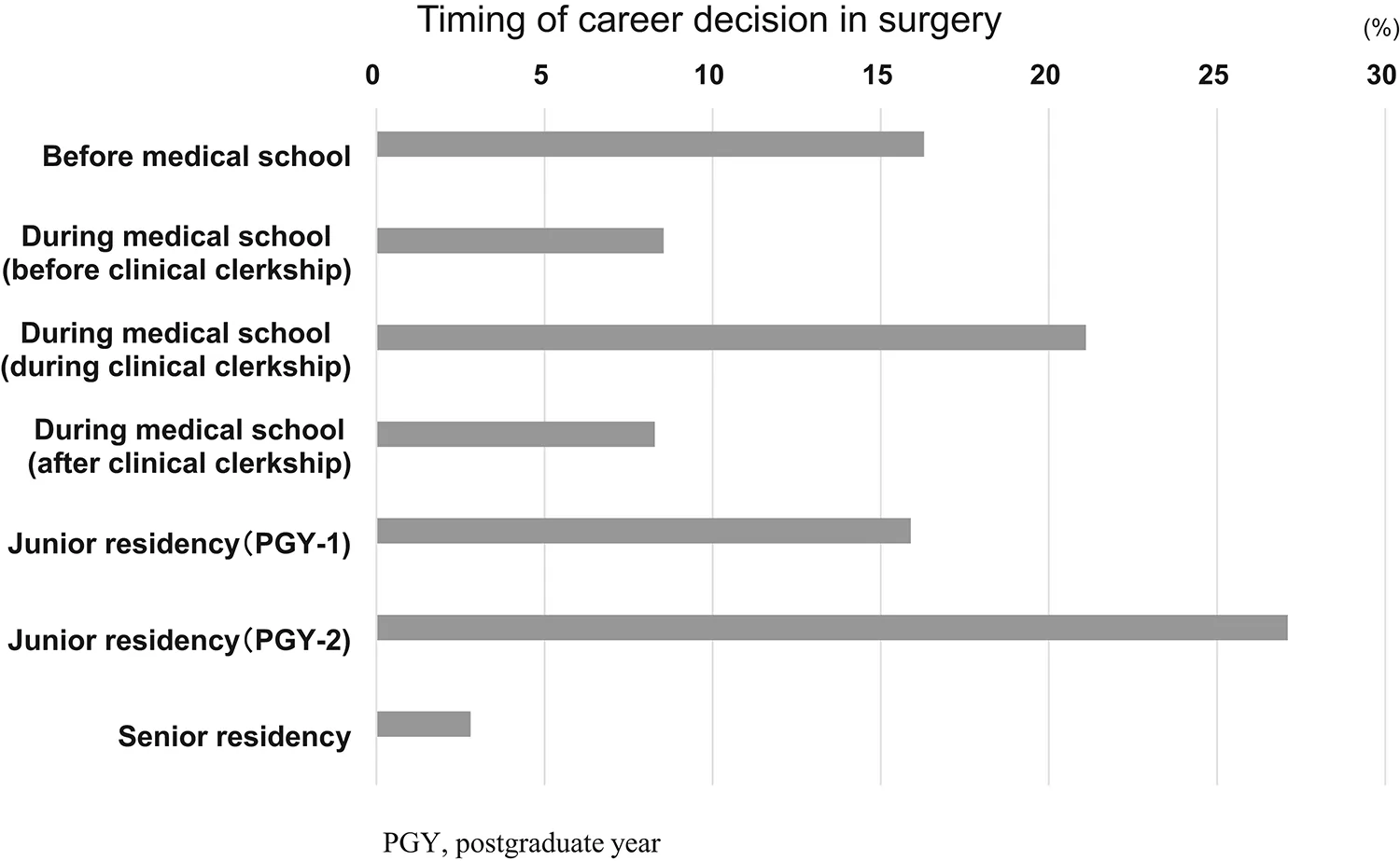
Timing of career decision in surgery among surgical residents. Bar chart showing the distribution of career decision timing among surgical residents in Japan. Decisions were most commonly made during junior residency (PGY-2), followed by a clinical clerkship in medical school and before entering medical school. PGY, postgraduate year

**Fig. 3:**
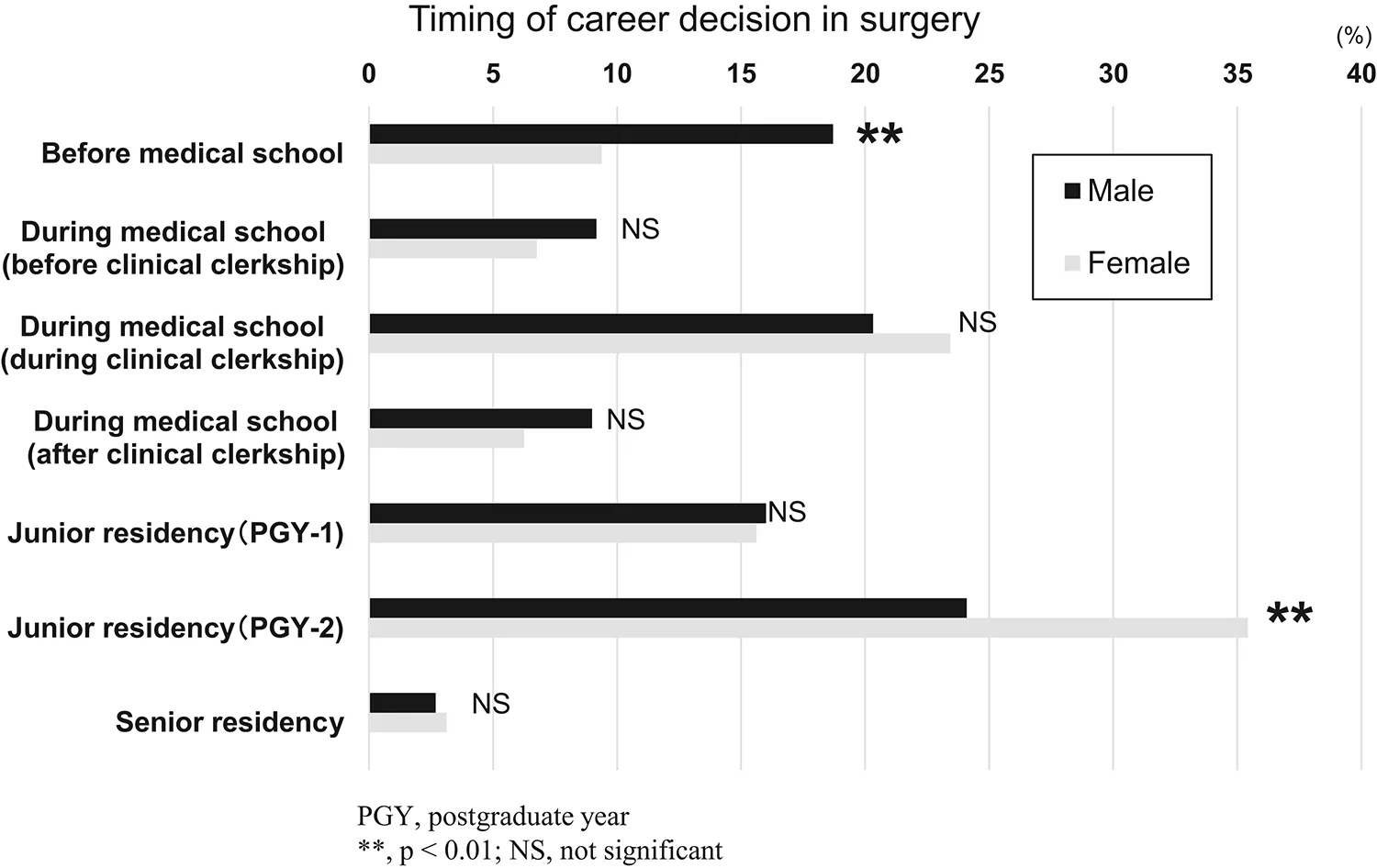
Gender differences in the timing of career decision in surgery. Bar chart comparing the timing of career decisions between male and female surgical residents. Male residents were more likely to decide on their specialty before entering medical school, while female residents more frequently chose surgery during PGY-2. Significant sex differences were observed in decisions made before medical school (*p* = 0.003) and during PGY-2 (*p* = 0.002). **p < 0.01; NS, not significant; PGY, postgraduate year

Most residents decided to become surgeons during junior residency (PGY-2, 27.1%), followed by clinical clerkship (21.1%), before medical school (16.3%), and junior residency (PGY-1, 15.9%) (Fig. 2 and Online Resource 1). Male residents were significantly more likely to decide to pursuing a surgical career before entering medical school than female residents (18.7% vs. 9.4%, *p* = 0.003) (Fig. 3 and Online Resource 2). In contrast, although the most common timing for this decision was during PGY-2 for both sexes, a significantly higher proportion of women than men chose a surgical career at this stage (35.4% vs. 24.1%, *p* = 0.002).

### Motivations for pursuing a surgical career

Among the eight major factors, surgery and procedures, the image of surgeons, and recruitment from surgeons were identified as positive influences on surgical-career decisions across all seven stages on the path to becoming a surgeon. Conversely, QOL and WLB negatively influenced all stages, followed by salary in five stages, the educational system and the process of becoming an independent surgeon in four stages, and career path in four stages (Online Resource 1).

The BJR group had a more positive impression of their decision-making regarding a surgical career in terms of the image of surgeons (65.8% vs. 56.0%, *p* = 0.016) and family or relatives (18.2% vs. 9.7%, *p* = 0.004) (Table 2). A gender-specific analysis revealed that among female surgeons in the BJR group, motivations for pursuing surgery were more influenced by the image of surgeons (55.2% BJR vs. 41.7% DJR, *p* = 0.048) and family or relatives (18.4% BJR vs. 8.8% DJR, *p* = 0.034) (Table 3). In contrast, female surgeons in the DJR group were more influenced by recruitment from surgeons (66.7% DJR vs. 47.1% BJR, *p* = 0.007) (Table 3). Among male surgeons in the BJR group, only familial influences were a prominent positive factor in their decision-making (18.1% BJR vs. 10.2% DJR, *p* = 0.041), whereas no significant differences were observed for the other factors (Table 4).

**Table 2:** Motivations affecting the decision-making for pursuing a surgical career stratified by timing: before and during or after junior residency

Factors	BJR group (n = 406)			DJR group (n = 343)			*p*
	Positive	Neutral	Negative	Positive	Neutral	Negative	
Surgery and procedures	367 (91.1)	28 (6.9)	8 (2.0)	294 (86.2)	41 (12.0)	6 (1.8)	0.059
Image of surgeons	265 (65.8)	122 (30.3)	16 (4.0)	191 (56.0)	127 (37.2)	23 (6.7)	0.016
Recruitment from surgeons	223 (55.3)	157 (39.0)	23 (5.7)	215 (63.2)	114 (33.5)	11 (3.2)	0.052
Educational system/ Process of becoming an independent surgeon	78 (19.5)	223 (55.9)	98 (24.6)	71 (21.0)	176 (52.1)	91 (26.9)	0.582
Family or relatives	73 (18.2)	275 (68.4)	54 (13.4)	33 (9.7)	249 (73.5)	57 (16.8)	0.004
Career path	60 (14.9)	243 (60.4)	99 (24.6)	34 (10.0)	205 (60.5)	100 (29.5)	0.078
Salary	29 (7.2)	259 (64.6)	113 (28.2)	29 (8.6)	231 (68.1)	79 (23.3)	0.295
QOL, WLB	15 (3.7)	141 (35.1)	246 (61.2)	12 (3.5)	101 (29.7)	227 (66.8)	0.280

Data are shown as n (%). BJR: residents who chose a surgery career before junior residency. DJR: residents who chose a surgery career during or after junior residency; QOL, quality of life; WLB, work-life balance

**Table 3:** Motivations affecting the decision-making for pursuing a surgical career among female residents

Factors	Female residents in BJR group (n = 88)			Female residents in DJR group (n = 104)			*p*
	Positive	Neutral	Negative	Positive	Neutral	Negative	
Surgery and procedures	83 (94.3)	4 (4.5)	1 (1.1)	92 (89.3)	9 (8.7)	2 (1.9)	0.216
Image of surgeons	48 (55.2)	33 (37.9)	6 (6.9)	43 (41.7)	47 (45.9)	13 (12.6)	0.048
Recruitment from surgeons	41 (47.1)	40 (46.0)	6 (6.9)	68 (66.7)	30 (29.4)	4 (3.9)	0.007
Educational system and process of becoming an independent surgeon	16 (18.6)	35 (40.7)	35 (40.7)	21 (20.8)	44 (43.6)	36 (35.6)	0.493
Family or relatives	16 (18.4)	59 (67.8)	12 (13.8)	9 (8.8)	71 (69.6)	22 (21.6)	0.034
Career path	8 (9.1)	45 (51.1)	35 (39.8)	8 (7.8)	51 (50.0)	43 (42.2)	0.699
Salary	3 (3.4)	64 (73.6)	20 (23.0)	8 (7.9)	73 (72.3)	20 (19.8)	0.321
QOL, WLB	3 (3.4)	21 (24.1)	63 (72.4)	4 (3.9)	19 (18.4)	80 (77.7)	0.434

Data are shown as n (%). BJR: residents who chose a surgery career before junior residency; DJR: residents who chose a surgery career during or after junior residency; QOL, quality of life; WLB, work-life balance

**Table 4:** Motivations affecting the decision-making for pursuing a surgical career among male residents

Factors	Male residents in BJR group (n = 318)			Male residents in DJR group (n = 238)			*p*
	Positive	Neutral	Negative	Positive	Neutral	Negative	
Surgery and procedures	284 (90.2)	24 (7.6)	7 (2.2)	201 (84.8)	32 (13.5)	4 (1.7)	0.066
Image of surgeons	217 (68.3)	89 (28.2)	10 (3.2)	148 (62.4)	79 (33.3)	10 (4.2)	0.122
Recruitment from surgeons	182 (57.6)	117 (37.0)	17 (5.4)	147 (62.0)	83 (35.0)	7 (3.0)	0.222
Educational system and process of becoming an independent surgeon	62 (19.8)	188 (60.1)	63 (20.1)	50 (21.2)	131 (55.5)	55 (23.3)	0.743
Family or relatives	57 (18.1)	216 (68.6)	42 (13.3)	24 (10.2)	177 (75.0)	35 (14.8)	0.041
Career path	52 (16.6)	198 (63.1)	64 (20.4)	26 (11.0)	154 (65.3)	56 (23.7)	0.089
Salary	26 (8.3)	195 (62.1)	93 (29.6)	21 (8.9)	158 (66.7)	58 (24.5)	0.223
QOL, WLB	12 (3.8)	120 (38.1)	183 (58.1)	8 (3.4)	82 (34.7)	146 (61.9)	0.374

Data are shown as n (%). BJR: residents who chose a surgery career before junior residency; DJR: residents who chose a surgery career during or after junior residency; QOL, quality of life; WLB, work-life balance

## Discussion

In this study, we analyzed the responses of recently board-certified surgeons to clarify the time at which they decided to pursue a surgical career and the factors that motivated them. Respondents were classified into two groups—those who made their decision before entering junior residency and those who decided during or after junior residency—and were analyzed accordingly. Regardless of timing, the strongest motivating factor for pursuing a surgical career was the experience of performing surgery and procedures. However, several background differences were observed between the two groups, and notable sex-specific differences were identified regarding decision timing and motivation.

Male residents were more likely to decide on a surgical career before entering medical school, whereas a significantly higher proportion of female residents made this decision during their second postgraduate year (PGY-2). This tendency for female residents to decide on a surgical career later than their male counterparts may be attributed to several gender-related gaps. First, women more frequently prioritize work-life balance and parental leave [5, 13–15]. These considerations may make them more cautious about committing to a surgical career until they are confident that adequate support systems are available in the workplace. In the 2013 survey, while approximately half of Japanese female surgeons felt that men were privileged at home, they also placed significant value on women’s roles within the family [16]. This can explain why a higher proportion of female residents chose surgery during PGY-2 after they had directly observed the clinical environment and assessed whether it aligned with their expectations and needs.

Second, the global shortage of role models for female surgeons presents a challenge, as female trainees often have limited access to same-gender mentorship, making it more difficult for them to envision themselves in surgical careers [17–21]. Although previous studies, including a qualitative study from Canada [22], have shown that gender influences interest in surgical careers, with women often delaying their decision because of lifestyle concerns, limited mentorship, or perceived gender bias, none have quantitatively compared the timing of such decisions between male and female trainees. To our knowledge, this is the first study to provide quantitative evidence of a sex-based difference in the actual stage of training at which the decision to pursue surgery is made.

The DJR group included more female residents and individuals from urban areas than the BJR group, reflecting the actual differences in training environments between urban and rural settings. Residents from rural backgrounds were more likely to pursue surgery early, as they were more influenced by the image of surgeons and family related motivations, which contributed to their earlier career decisions. One possible explanation is that trainees from non-urban backgrounds may encounter a relatively narrower range of visible career options, which may facilitate an earlier commitment to surgery. In addition, in more closed local environments, interpersonal relationships may have a stronger influence on career decision-making. In contrast, urban trainees are likely to be exposed to greater diversity in career paths and more frequent movement of people across institutions, which may broaden their options and delay career decisions. In addition, female residents in the DJR group were more influenced by direct recruitment from the surgeons. Being directly approached by surgeons may serve as a signal that they are welcomed into the surgical community, thereby fostering psychological safety. Such encouragement may also help alleviate concerns regarding WLB and career continuity and make it easier for trainees to envision surgery as a feasible long-term career. This suggests that female surgeons’ concerns about balancing surgical practice with childbirth and parenting may influence the timing of their career decisions, and that direct encouragement from surgeons may help alleviate such concerns. Among male residents, family influence was a more prominent factor in the BJR group than in the DJR group, further suggesting that personal backgrounds and support networks can shape career decisions differently based on training routes.

Providing opportunities to enhance the perception of surgery during medical school, such as early exposure to surgical practice or mentorship from inspiring surgeons, may be particularly effective in recruiting male medical students. Proactive encouragement from surgical departments during clinical rotations, along with addressing common concerns such as work-life balance and training intensity, may be especially beneficial for recruiting female residents in the future. These targeted strategies may strengthen the recruitment and retention of a diverse and dedicated surgical workforce in the future.

This study has several limitations. The key strength of this survey is its near-census design: we queried the entire cohort of candidates for the Japanese surgical board examination, thereby minimizing selection bias and strengthening generalizability. The response rate of 54% may have caused some selection bias, as the results might not reflect the views of non-responding surgeons among all newly board-certified surgeons. Furthermore, because this survey included only surgical trainees, it did not capture the perspectives or decision-making factors of individuals who may have considered surgery during their PGY-2 but ultimately chose another specialty. This limits the strategic interpretation of factors relevant to recruitment among undecided or nonsurgical trainees. A questionnaire survey targeting medical students or doctors who have not yet decided on a specialty could be helpful in capturing authentic perspectives, thereby clarifying which aspects of the surgical career path should be emphasized or reconsidered.

In conclusion, these findings underscore the importance of considering individual backgrounds and gender when developing strategies to attract and retain talented surgical trainees. Enhancing early exposure opportunities, providing relatable role models, and addressing gender-specific motivational factors may help cultivate a diverse and sustainable surgical workforce. By highlighting gender differences in the timing and motivations for pursuing a surgical career, this study suggests that understanding when and how to appeal to potential recruits, particularly women, can significantly improve future recruitment strategies.

## Electronic Supplementary Material

Below is the link to the electronic supplementary material.

Supplementary Material 1
