# Supplementary material for: Timing and motivation behind the decision to become a surgeon: Gender and optimal recruitment insights
Source: Surg Today. 2026 Jun 15;56(10):2311–8. doi: 10.1007/s00595-026-03361-6 (PMC13558389; doi:10.1007/s00595-026-03361-6)
Supplement: Supplementary file 1 [file 595_2026_3361_MOESM1_ESM.docx]

| Online Resource 1. Reasons for choosing a career in surgery stratified by career stages | | | | | | | | | | | | | | | | | | | | | | | | | | |
| --- | --- | --- | --- | --- | --- | --- | --- | --- | --- | --- | --- | --- | --- | --- | --- | --- | --- | --- | --- | --- | --- | --- | --- | --- | --- | --- |
| Career stages  Factors | Before medical school | | | During medical school  (before clinical clerkship) | | | During medical school  (during clinical clerkship) | | | During medical school  (after clinical clerkship) | | | | Junior residency (PGY-1) | | | | Junior residency (PGY-2) | | | | Senior residency | | | |  |
|  | 122 (16.3) | | | 64 (8.5) | | | 158 (21.1) | | | 62 (8.3) | | | | 119 (15.9) | | | | 203 (27.1) | | | | 21 (2.8) | | | |  |
|  | Positive | Neutral | Negative | Positive | Neutral | Negative | Positive | Neutral | Negative | Positive | Neutral | Negative | Positive | | Neutral | Negative | Positive | | Neutral | Negative | Positive | | Neutral | Negative |  |  |
| Surgery and procedures | 105 (86.1) | 12 (9.8) | 4 (3.3) | 58 (90.6) | 6 (9.4) | 0 | 146 (92.4) | 10 (6.3) | 1 (0.6) | 58 (93.5) | 0 | 3 (4.8) | 112 (94.1) | | 6 (5.0) | 0 | 165 (81.3) | | 32 (15.8) | 5 (2.5) | 17 (81.0) | | 3 (14.3) | 1 (4.8) |  |  |
| Image of surgeons | 94 (77.0) | 21 (17.2) | 6 (4.9) | 40 (62.5) | 23 (35.9) | 1 (1.6) | 94 (59.5) | 58 (36.7) | 4 (2.5) | 37 (59.7) | 20 (32.3) | 5 (8.1) | 76 (63.9) | | 39 (32.8) | 3 (2.5) | 103 (50.7) | | 82 (40.4) | 17 (8.4) | 12 (57.1) | | 6 (28.6) | 3 (14.3) |  |  |
| Recruitment by surgeons | 57 (46.7) | 55 (45.1) | 10 (8.2) | 34 (53.1) | 23 (35.9) | 6 (9.4) | 97 (61.4) | 54 (34.2) | 6 (3.8) | 35 (56.5) | 25 (40.3) | 1 (1.6) | 82 (68.9) | | 32 (26.9) | 4 (3.4) | 125 (61.6) | | 70 (34.5) | 6 (3.0) | 8 (38.1) | | 12 (57.1) | 1 (4.8) |  |  |
| Educational system and process of becoming an independent surgeon | 27 (22.1) | 57 (46.7) | 37 (30.3) | 12 (18.8) | 40 (62.5) | 11 (17.2) | 26 (16.5) | 93 (58.9) | 35 (22.2) | 13 (21.0) | 33 (53.2) | 15 (24.2) | 34 (28.6) | | 58 (48.7) | 25 (21.0) | 35 (17.2) | | 106 (52.2) | 59 (29.1) | 2 (9.5) | | 12 (57.1) | 7 (33.3) |  |  |
| Family or relatives | 40 (32.8) | 66 (54.1) | 15 (12.3) | 7 (10.9) | 48 (75.0) | 8 (12.5) | 21 (13.3) | 117 (74.1) | 19 (12.0) | 5 (8.1) | 44 (71.0) | 12 (19.4) | 11 (9.2) | | 88 (73.9) | 18 (15.1) | 21 (10.3) | | 147 (72.4) | 33 (16.3) | 1 (4.8) | | 14 (66.7) | 6 (28.6) |  |  |
| Career path | 23 (18.9) | 67 (54.9) | 31 (25.4) | 12 (18.8) | 35 (54.7) | 16 (25.0) | 23 (14.6) | 98 (62.0) | 36 (22.8) | 2 (3.2) | 43 (69.4) | 16 (25.8) | 16 (13.4) | | 77 (64.7) | 24 (20.2) | 14 (6.9) | | 116 (57.1) | 71 (35.0) | 4 (19.0) | | 12 (57.1) | 5 (23.8) |  |  |
| Salary | 10 (8.2) | 66 (54.1) | 45 (36.9) | 6 (9.4) | 45 (70.3) | 12 (18.8) | 8 (5.1) | 111 (70.3) | 37 (23.4) | 5 (8.1) | 37 (59.7) | 19 (30.6) | 6 (5.0) | | 86 (72.3) | 26 (21.8) | 22 (10.8) | | 128 (63.1) | 50 (24.6) | 1 (4.8) | | 17 (81.0) | 3 (14.3) |  |  |
| QOL, WLB | 8 (6.6) | 44 (36.1) | 69 (56.6) | 2 (3.1) | 21 (32.8) | 40 (62.5) | 4 (2.5) | 60 (38.0) | 93 (58.9) | 1 (1.6) | 16 (25.8) | 44 (71.0) | 6 (5.0) | | 45 (37.8) | 67 (56.3) | 5 (2.5) | | 51 (25.1) | 145 (71.4) | 1 (4.8) | | 5 (23.8) | 15 (71.4) |  |  |
| Data are shown as n (%). The top three positive factors are highlighted in light red, while the top three negative factors are shaded in light blue. PGY, postgraduate year; QOL, quality of life; WLB, work-life balance | | | | | | | | | | | | | | | | | | | | | | | | | | |
|  | | | | | | | | | | | | | | | | | | | | | | | | | | |
|  | | | | | | | | | | | | | | | | | | | | | | | | | | |

| Online Resource 2. Timing of career decision in surgery stratified by sex | | | | |
| --- | --- | --- | --- | --- |
|  | Male | Female | | *p* |
| Career stage | n = 556 | n = 192 | |  |
| Before medical school | 104 (18.7) | 18 (9.4) | 0.003 | |
| During medical school (before clinical clerkship) | 51 (9.2) | 13 (6.8) | 0.310 | |
| During medical school (during clinical clerkship) | 113 (20.3) | 45 (23.4) | 0.360 | |
| During medical school (after clinical clerkship) | 50 (9.0) | 12 (6.3) | 0.240 | |
| Junior residency (PGY-1) | 89 (16.0) | 30 (15.6) | 0.900 | |
| Junior residency (PGY-2) | 134 (24.1) | 68 (35.4) | 0.002 | |
| Senior residency | 15 (2.7) | 6 (3.1) | 0.760 | |
| Data are shown as n (%). |  |  | |  |
